# Fibrin glue pleurodesis for pneumothorax in extremely preterm infants: a case report and literature review

**DOI:** 10.1186/s13052-018-0533-6

**Published:** 2018-08-14

**Authors:** Livia Drovandi, Ilaria Cianchi, Simone Pratesi, Carlo Dani

**Affiliations:** 1 Division of Neonatology, Careggi University Hospital of Florence, Largo Brambilla 3, 50141 Florence, Italy; 2 Department of Neurosciences, Psychology, Drug Research and Child Health, Careggi University Hospital of Florence, Florence, Italy

**Keywords:** Pneumothorax, Fibrin glue, Preterm infant

## Abstract

**Background:**

Chest tube drainage and mechanical ventilation are effective treatment of symptomatic pneumothorax (PTX), but the best management of persistent (> 7 days) PTX is unknown.

**Case presentation:**

We reported a case of successful fibrin glue pleurodesis of persistent PTX in an extremely preterm infant without adverse effects. We discussed previous literature on this treatment.

**Conclusions:**

Overall, the twelve reported cases suggest that persistent PTX sealing with fibrin glue can represent a simple, quick, and effective treatment whose possible reported adverse effects are transient and do not cause permanent sequelae. Thus, fibrin glue pleurodesis might be considered a suitable therapeutic tool in very preterm infant with persistent PTX.

## Background

Pneumothorax (PTX) is one of the major complications in extremely preterm infants with respiratory failure and is associated to high mortality and morbidity [1, 2]. After the introduction of surfactant replacement therapy, its incidence is dropped and now is around 1–2% [1, 2]. Asymptomatic PTX without underlying pulmonary disease does not require any treatment, but in case of symptomatic PTX insertion of a chest tube and air drainage are required [1]. Although necessary, chest tube placement can lead to several complications in neonates, such as lung injuries, insertion site and pulmonary infections, phrenic nerve paralysis, chylothorax, and hemorrhagic pericardial effusion [1]. Usually chest tube drainage, positioning with the affected side down, low lung volume strategy, and high frequency oscillatory ventilation are effective treatment of PTX and allow its recovery in few days but, sometimes, mainly in patients with large bronchopleural fistulas, it can persist for several days [2].

Bhatia and Mathew proposed that PTX longer than 7 days should be defined as persistent PTX [3]. They demonstrated that preterm infants with persistent PTX have higher risk of developing chronic lung disease than infants in whom PTX resolved before [3].

Unfortunately, there are not guidelines indicating the most appropriate approach to persistent PTX before considering more invasive procedures**,** such as pleurectomy, chemical pleurodesis, and selective intubation of the contralateral bronchus [4]. On the other hand, the risk of associated morbidities, such as intracranial hemorrhage, acute hypotension, air embolus and circulatory failure, urges the treatment of persistent PTX [2].

Fibrin glue is a two-component material consisting of concentrated human fibrinogen and thrombin imitating the coagulation process of the body. By combining the thrombin and fibrinogen component conversion of fibrinogen to fibrin takes place and a clot is formed by a three-dimensional fibrin network [5]. Fibrin glue on-label indications in adults include hemostasis, colonic sealing, and skin graft attachment [6]. Recent clinical and experimental uses include tissue or mesh attachment, fistula closure, lymphatic sealing, adhesion prevention, drug delivery, and tissue engineering [6].

The first case of successful fibrin glue pleurodesis to seal a persistent PTX in a preterm infant has been reported in 1993 by Berger et al. [7]. From then, other case reports have been published [1, 2, 8, 9] suggesting that this procedure is effective in sealing persistent PTX in these patients without inducing severe adverse effects.

Thus, the purpose of this report is to document a further case of fibrin glue pleurodesis of persistent PTX in an extremely preterm infant, and to discuss previous literature on this issue for drawing the attention of Neonatologists to this little-known promising treatment.

## Case presentation

**Fig. 1:**
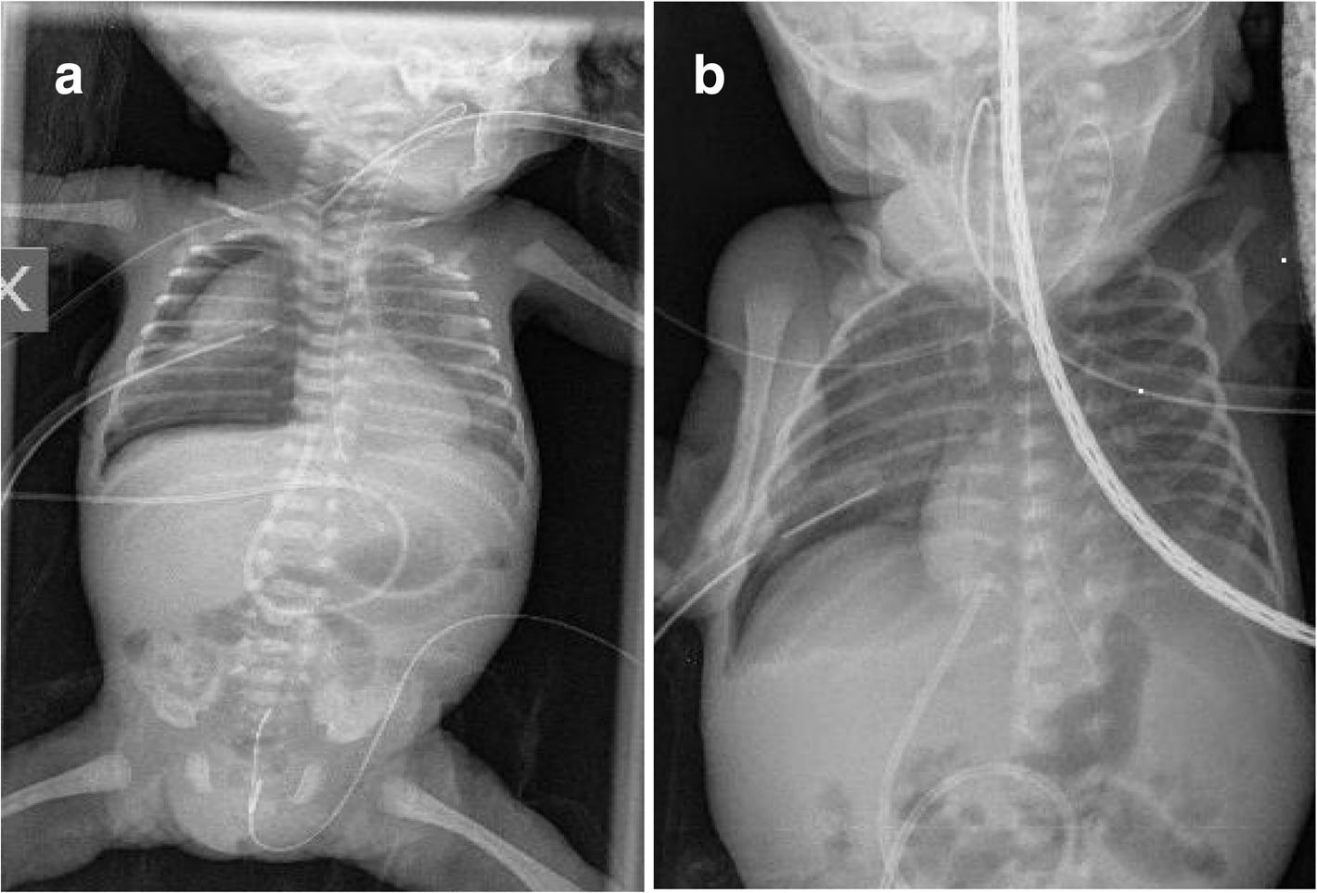
PTX in the first and fourteenth day of life. Chest x-ray showing right-sided PTX in the first day of life (**a**) persisting in the fourteenth day of life (**b**)

A male was born at 26 weeks of gestation by cesarean section due to maternal vaginal bleeding after antenatal steroids. His birth weight was 810 g and Apgar scores were 7 at 1 min and 8 at 5 min. Non-invasive mechanical ventilation (N-IMV with the following parameters: peak inspiratory pressure 18 cm H_2_O, respiratory rate 60/min, positive end expiratory pressure 5 cm H_2_O, FiO_2_ 0.35) was started immediately after delivery because of respiratory distress and continued in neonatal intensive care unit (NICU). Our patient was treated with surfactant (200 mg/kg of Curosurf, Chiesi Farmaceutici Spa, Parma, Italy) with the InSURE (Intubation-SURfactant-Extubation) procedure at 2 h of life when FiO_2_ was 0.40. At 24 h of life a second dose of surfactant (100 mg/kg) was given (again with the InSURE procedure) for the worsening of respiratory failure (FiO_2_ 0.45), but immediately after the procedure he developed a right-sided PTX (Fig. 1). Therefore, high frequency oscillatory ventilation (HFOV) was started and chest tube was placed on 10 cm H_2_O suction for continuous pleural drainage. On the fourteenth day of life the PTX persisted (Fig. 1) and, obtained informed parental consent, we performed chest pleurodesis with fibrin glue.

**Fig. 2:**
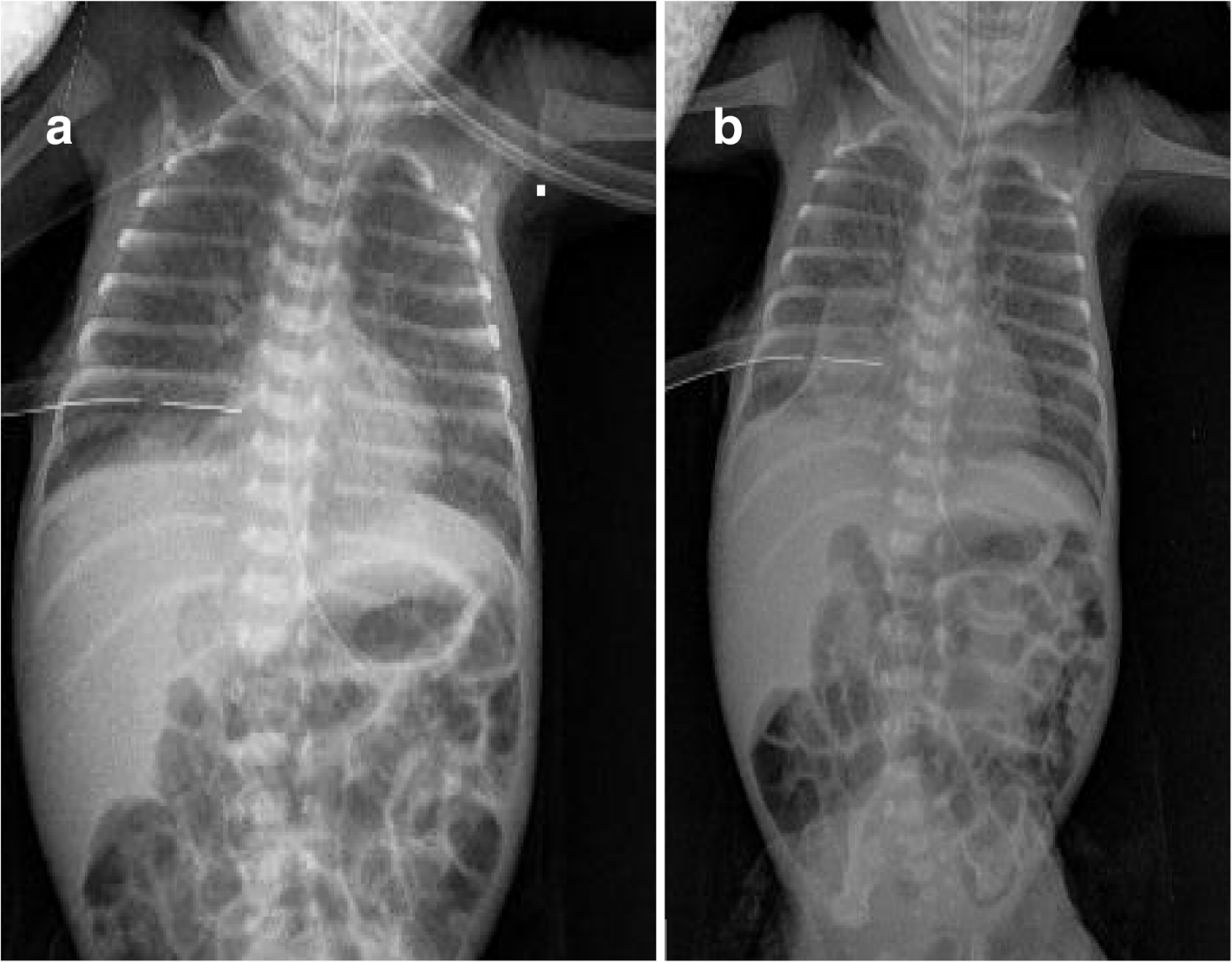
Chest x-ray after fibrin glue injection. Chest x-ray at 30 min (**a**) and 12 h (**b**) after fibrin glue injection. A minimal PTX was appreciable in (**a**) and disappeared in (**b**). At the distal tip of the chest tube is visible a radiopaque area referable to the fibrin glue clot more evident in (**b**) than in (**a**)

**Fig. 3:**
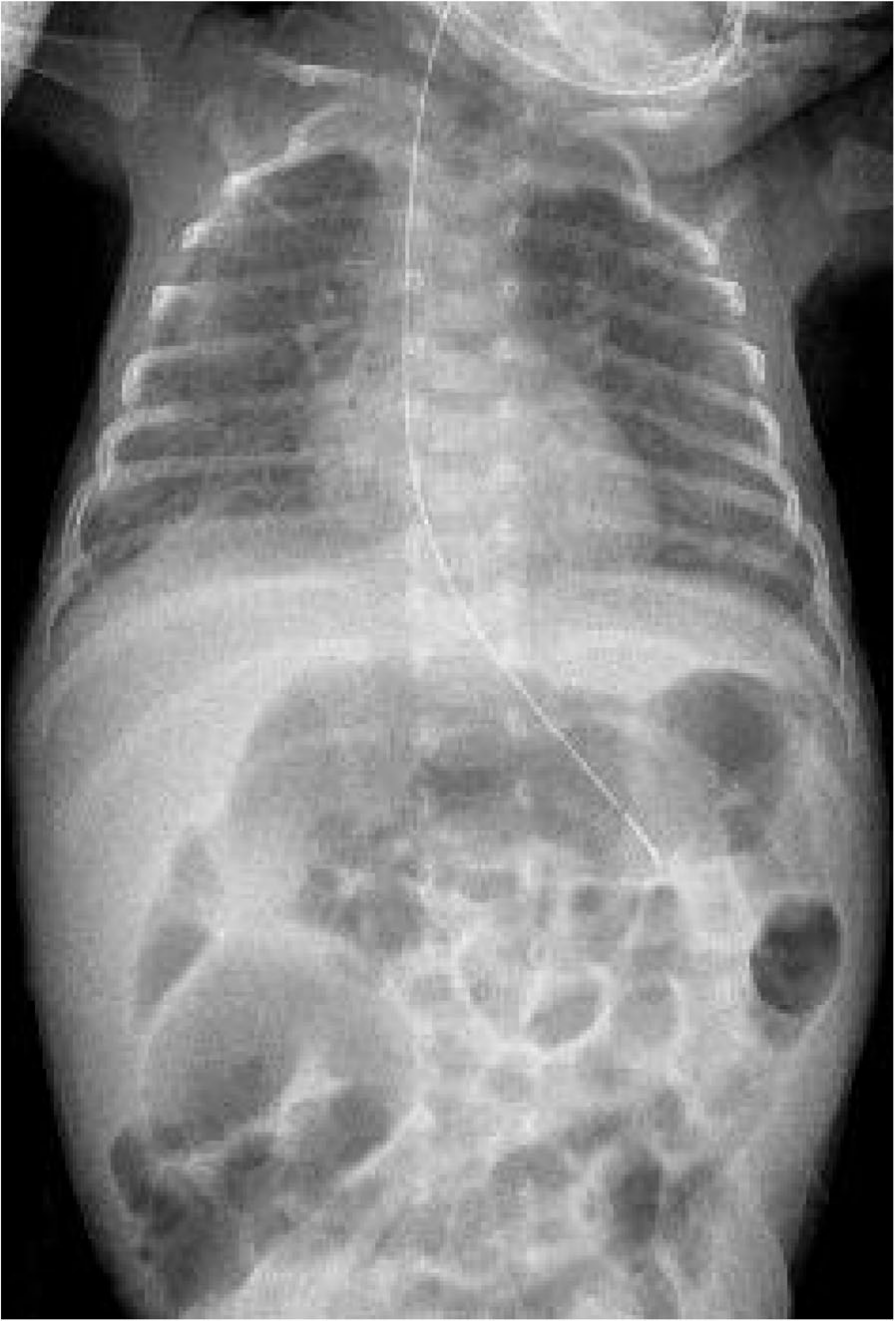
Chest x-ray at discharge. Chest x-ray at 78 day of life showing the disappearance of the fibrin glue

The homologous fibrin glue (Tisseel®, Baxter, Lurago d’Erba, Italy) was prepared in a prefilled double chamber syringe which contains sealer protein solution (with synthetic aprotinin) in one chamber and thrombin solution (with calcium chloride) in the other chamber resulting in 10 ml of product ready for use. The administration procedure was similar to previous reports [1, 2, 7–9]: a 5-Fr umbilical catheter was placed into the 10F chest tube during HFOV and 5 mL of fibrin were slowly injected into the right pleural space. After the tube was completely pulled by the glue, the catheter was slowly extracted. During the 5 min injection heart rate was stable, and SpO_2_ was > 90% with unchanged FiO_2_. The procedure was followed by a quick and marked reduction in airflow through the chest tube and chest X-ray performed 30 min later showed a minimal right PTX (Fig. 2). After 12 h, no air outflow was visible through the chest tube and PTX disappeared at chest X-ray (Fig. 2); 24 h later the tube was clamped and 48 h after the fibrin glue pleurodesis it was definitely removed. Five days later mechanical ventilation was stopped, and non-invasive ventilatory support was finally interrupted on 78 day of life when chest X-ray demonstrated the disappearance of fibrin glue clot (Fig. 3).

## Discussion

The management of persistent PTX is not well established but prolonged chest tube placement and mechanical ventilation certainly increase the risk of developing severe complications (i.e.: infections, hemodynamic disturbances, bronchopulmonary dysplasia, etc.), especially in the subset of preterm infant population. Therefore, a relatively simple and quick procedure such as the fibrin glue pleurodesis may be very appealing for the treatment of persistent PTX.

Previous studies reported eleven cases of persistent PTX sealing with fibrin glue in very preterm infants [1, 2, 7–9] (Table 1). As in our case, fibrin glue pleurodesis was successful in all survived patients in whom PTX resolved within 48 h from the treatment as demonstrated by control chest x-rays [1, 2, 7–9]. The dose of fibrin glue varied from 3.5 [2] to 10 mL [7], in relationship to different types of fibrin glue and different injection devices. In the majority of cases a single course of fibrin glue was effective [1, 2, 7–9], but in two cases of recurrent PTX a second course was successfully performed [8]. Moreover, one patient had bilateral persistent PTX and received successful bilateral treatment [1]. Adverse effects were associated to the procedure in 5 patients in a single study [8], including bradycardia, hypercalcemia, skin necrosis, and diaphragmatic paralysis. It has been speculated that most of the complications (bradycardia, hypercalcemia, and the skin necrosis) were secondary to the concentration of calcium in the fibrin glue [8]. Nevertheless, these adverse effects did not occur in the remaining 7 patients (including ours) [1, 2, 7], and, in any case, they were transient and did not cause severe permanent sequelae [1, 2, 7–9]. However, the actual role of calcium is difficult to evaluate because different types of fibrin glue with different calcium content were used [1, 2, 7–9]. Possible effects of fibrin glue on patients’ long-term pulmonary function might be a source of concern, However, our case and previous studies showed that chest X-rays performed at discharge [7], or later [1, 9] did not evidence lung abnormalities referable to fibrin glue pleurodesis.

**Table 1:** Summary of reported cases of fibrin glue pleurodesis for persistent PTX in preterm infants

	Gestational age (wks)	Birth weight (g)	Number of cases	Age at treatment (d)	Fibrin glue dose (mL)	Complications
Berger [7]	26	850	1	30	10	–
Kuint [9]	31	–	1	59	5	–
Sarkar [8]	24–29	530–1500	7			Bradycardia, hypercalcemia, skin necrosis, transient diaphragm paralysis
Campolat [2]	25	790	1	15	3.5	–
Nishizaki [1]	25	764	1	8	6	–

Between possible treatments of persistent PTX, pleurectomy is too much invasive and, therefore, unfeasible in very preterm infants, while selective intubation of contralateral bronchus is very difficult particularly when the left main stem bronchus has to be selectively intubated. Pleurodesis with povidone-iodine has been reported in refractory chylotorax in newborns [10] and in a single-case of persistent PTX [4]. However, the use of povidone-iodine for pleurodesis can rise some concerns in very preterm infants due its chemical inflammatory effects, and the iodine oxidative and cytotoxic properties plus the risk of secondary hypothyroidisms [4, 10]. Moreover, the povidone-iodine pleurodesis in neonates has been associated with adverse effects, such as pain [11], lung or lobar collapse [11], and acute renal failure [12]. Conversely, fibrin glue is a human product that is catabolized by physiologic fibrinolytic agents whose reported adverse effects seem to be less severe.

## Conclusions

The best management of persistent PTX in very preterm infants has not been reported in literature and the appropriate duration of standard therapy (pleural drainage through a chest tube during mechanical ventilation) before considering more invasive procedures has not been established. We reported a case of persistent PTX in an extremely low birth-weight infant who was successfully treated with fibrin glue pleurodesis. Our and previous case reports [1, 2, 7–9] suggest that persistent PTX sealing with fibrin glue can represent a simple, quick, and effective treatment whose possible reported adverse effects are transient and do not cause permanent sequelae. Thus, we believe that fibrin glue pleurodesis might be considered a suitable therapeutic tool in very preterm infant with persistent PTX.

## Abbreviations

HFOV: High frequency oscillatory ventilation
InSURE: Intubation-SURfactant-Extubation
N-IMV: Non-invasive mechanical ventilation
PTX: Pneumothorax
